# Cystinuria: An Overview of Challenges and Surgical Management

**DOI:** 10.3389/fsurg.2022.812226

**Published:** 2022-06-16

**Authors:** Calum Stephen Clark, Sanjith Gnanappiragasam, Kay Thomas, Matthew Bultitude

**Affiliations:** Guy’s and St Thomas’ NHS Foundation Trust, London, United Kingdom

**Keywords:** endourology, calculi, ureteroscopy, PCNL, cystinuria, ESWL (Extracorporeal Shock Wave Lithotripsy), ECIRS

## Introduction

Cystinuria is a genetically inherited condition and a rare cause of kidney stones. It affects approximately 1 in 7,000 of the global population, although wide geographical variances exist (1). It is often quoted that cystine stones make up 1–2% of all urinary stones in adults and 6–8% in pediatric populations (2). Cystinuria is typically thought of as an autosomal recessive disease but can be autosomal dominant with incomplete penetrance (1, 3). It is caused by a defective amino acid transporter in the proximal renal tubules and in the epithelial cell lining of the small intestine affecting transport of cystine and the dibasic amino acids ornithine, lysine, and arginine (COLA). Cystine is relatively insoluble (compared with the other three amino acids), and thus, cystine can precipitate out, causing renal stone formation. The responsible genetic defects are located in genes SLC3A1 (2p21) and SLC7A9 (19q12), which encode the cystine transporter (3). Historically, patients were classified by the levels of urinary cystine excretion, but a more recent genotype classification is now used with Type A (mutations in SLC3A1), Type B (mutations in SLC7A9), or Type AB (1 mutation in each gene) (4).

Most patients present with the stone disease before age 30 years with the peak incidence between 11 and 20 years (1, 5, 6). Patients often suffer from lifelong stone formation, although the phenotype varies from mild (no stones) to severe (highly recurrent). As a consequence of recurrent stone episodes and interventions, chronic kidney disease and hypertension are common, and cystine stone formers have been shown to have worse health-related quality of life compared to noncystine stone formers (3, 7).

Due to the highly recurrent nature of kidney stones, cystinuria can pose significant diagnostic, logistical, and surgical challenges.

## Challenges of Cystinuria

### Frequent Stone Formation

Typically, cystine stone formers produce stones at a faster rate and from an earlier age than noncystine stone formers (8). Streeper et al. looked at cystinuria patients’ overall quality of life and found that stones formed in this cohort, on average, every 12–24 months, with the typical patient having undergone up to seven endourological procedures by middle age (9). In comparison, noncystinuria patients have an overall lifetime risk of urolithiasis of between 10 and 15% and a 10-year risk of recurrence of around 50% (10). In our experience of running a specialist cystinuria clinic, we have found that 83% of patients presented with their first stone before the age of 30 years (5). Moreover, due to the relatively rare nature of cystinuria, up to a quarter had a delayed diagnosis, with an average time from initial presentation to diagnosis of 7.8 years (5). This delay in diagnosis can result in irreversible kidney damage due to repeated renal insult or from continuous or undetected stone disease (5). In total, 8% of our patient cohort had already undergone a nephrectomy, secondary to stone disease, by the time they were seen in our clinic (5). This highlights the importance of prompt recognition and diagnosis of the condition so treatment can be initiated to slow stone formation, with an overall aim to preserve renal function (3, 5, 9, 11).

#### Dietary Advice for Cystine Stone Formers

The cornerstone of dietary advice for patients with cystinuria is to ensure a high fluid intake aiming for a minimum urine output of 3 L/day (3, 6). This fluid intake should ideally be spread out throughout the day, and in extreme cases, patients are advised to wake at night to drink fluid (3). The aim of this is to reduce the concentration of cystine to <250 mg/L in the urine to prevent crystallization. Diet is important, and patients should be advised to follow a low-salt diet as this has been shown to reduce cystine excretion (8). Restriction of animal protein is also recommended to limit the intake of methionine (a precursor of cystine) (3). Often dietary modifications are not enough on their own, and alkalinization of the urine with potassium citrate is recommended to achieve a urine pH of 7.5 (3, 6). We recommend that patients periodically check their urine pH at different times of the day to assess whether this target is achieved. In patients who continue to form stones despite the above advice and urinary alkalinization, a cystine-binding thiol drug such as tiopronin or D-penicillamine is used (3, 6). Such a drug binds cystine and results in a drug-cysteine complex that is up to 50 times more soluble than cystine. Both these medications can have significant side effects and require regular urinary and serum monitoring for proteinuria and blood counts.

### Compliance with Diet and Medications and Engagement with Health Services

Cystinuria typically affects a young cohort of patients (1, 2). Prevention of stone formation is the main objective in cystinuria, aiming to reduce the level of urinary cystine to below the point of solubility that stops crystal formation (13). As discussed, patients are initially trialed with conservative management, which includes dietary modification and fluid intake in excess of 3 L/day, to avoid crystal excretion and aggregation in the urinary tract (14). Medication can be introduced if conservative management alone is not sufficient to control stone formation (15). Adherence to both conservative and medical management can be a hurdle for patients who may require support from urologists, nephrologists, and dieticians to achieve long-term goals (16). Dietary adaptation can be restrictive for patients, as can the requirement to drink in excess of 3 L/day of water for practical reasons. The importance of engagement with medical management and services must be emphasized to patients to ensure they are minimizing their risk of stone formation and surgical intervention while also being monitored and treated for other complications such as chronic kidney disease and hypertension (16, 17).

To maintain satisfactory levels of urinary cystine and reduce the rate of stone formation, a close long-term relationship with the multidisciplinary team managing the condition is key (5). Medical management may need to be closely monitored and adapted to the individual over time, and early stone detection is vital to ensure there is a minimal overall loss of renal function with increasing age (15). This relationship and continuity can be difficult to achieve, particularly through the transition from pediatric and adolescence into adult services, often requiring a change of the nephrologist and surgeon. In addition, geographical location can make this continuity logistically difficult. While finding a regime that is successful and suits the individual, multiple hospital visits are often required to monitor treatment effectiveness. Depending on services and expertise available locally, patients are often referred to tertiary centers to aid with the management of this condition. Depending on geographical location, the added burden of frequent long-distance commutes to centers that specialize in the treatment of cystinuria can take its toll. In our experience, providing a dedicated cystinuria clinic can help with service engagement by providing a one-stop clinic where patients can access all specialties involved in their specialty care during one visit.

### Side-Effects/Monitoring and Availability of Medications

For those who are unresponsive to both dietary modifications and increased oral fluid intake alone, pharmacological management may need to be initiated (5, 12, 14). Urinary alkalization with sodium bicarbonate or potassium citrate is the first-line medical treatment for those with unsatisfactory levels of urinary cystine and who still continue to make stones (5). As with conservative measures, strict adherence to medical therapy is required to be effective (5). These medications can be associated with unpleasant side effects including nausea and other gastrointestinal symptoms that can impact patient compliance (18, 19). Urinary pH and plasma potassium or sodium levels need to be closely monitored to ensure they are within satisfactory and safe ranges yet still sufficient to effectively reduce the rate of stone formation (14). Twenty-four-hour urinary cystine concentration can be used to monitor treatment effectiveness, and chelation therapy may be needed aiming for a urinary cystine concentration of <250 µmols/L (5).

Chelation therapy includes thiol-based agents, tiopronin or D-penicillamine (20), that can be added when other interventions have failed to halt stone formation (19, 20). Again, compliance with these medications is important and can remain an issue as they are also associated with a profile of significant side effects (20, 21). They require individual dosing regimes, particularly in the pediatric population that is calculated and adapted relative to body weight (14, 20). Toxicity and adverse sensitivity reactions can occur in up to 40% of children, which usually present with a rash, fever, or more rarely arthropathy (22). In the wider population, side effects can range from the mild, e.g., gastrointestinal upset, to more severe blood dyscrasias and nephrotic syndrome (15, 22, 23). More rarely, D-penicillamine can induce autoimmune reactions. The incidence of side effects was found to be dose-dependent, therefore making sure correct doses of medications are prescribed and adjusted accordingly through adolescence into adulthood (22).

One challenge with these medications is availability and cost, which varies across the world. In the United Kingdom, tiopronin is unlicensed and thus can only be prescribed in specialist centers and can be difficult to source, while penicillamine is not available in many countries. Both require close and long-term plasma and urinary monitoring (22). In addition, potassium citrate can be very expensive and is often not well tolerated (22).

### Prevalence of CKD/Hypertension

The renal insults from recurring stone formation, colic episodes, and interventions can result in damage to the kidneys, reduced overall renal function, and chronic kidney disease (CKD) (5, 17). In a large series from France, Prot-Bertoye et al. reported on 442 cystinuric patients in a retrospective study. In total, 77.5% had an abnormal e-GFR (<90 mLs/min) and 26.7% had e-GFR < 60. Among the patients with CKD, the incidence of hypertension was 28.6% (17). In our series, we reported a similarly high incidence with 75% having CKD, and hypertension was found in 50.8% of our series (16).

## Surgical Management

### Ureteric Stones

The clinical presentation of cystine stone disease is identical, although it can be more frequent than that of other stone compositions (5, 24). Patients may experience episodes of colic due to ureteric stones but can also present with renal pain, urinary infection, haematuria, or stones that can be picked up incidentally through routine imaging (3, 11). Those who have experienced colic pain before will often recognize the symptoms and may try to pass the stone without presenting for imaging or intervention. For these patients, often ultrasound may be enough as a first-line investigation to try and reduce the exposure to ionizing radiation (3, 6). However, if a stone is not passed or if there are persistent symptoms, then a low-dose noncontrast CT will be required.

Anecdotally, cystinuria patients often have more capacious ureters than noncystine stone formers (due to recurrent ureteric stones and multiple endourological interventions) and may be able to pass stones larger than would normally be expected compared to other composition stone formers. As for any stone former, obstruction in a single kidney or the presence of infection should prompt immediate assessment and intervention to relieve the obstruction and preserve overall renal health (2).

Due to the frequent nature of stone formation, cystinuria patients may know the size of stones they are historically able to pass, allowing for surveillance and conservative management of calculi smaller than this. However, if stones do not pass promptly or are unlikely to pass due to size or location, then timely intervention should be offered to prevent the potential loss of renal function from prolonged recurrent episodes of ureteric obstruction (5, 13). For obstructing ureteric stones, either extracorporeal shockwave lithotripsy (ESWL) or ureteroscopy (URS) can be offered depending on the knowledge of previous intervention success rates, availability, and patient preference, helping to guide the decision-making process. However, as cystine stones may not show up on plain imaging and as ureteroscopy offers a very high chance of clearing the stone and relieving the obstruction in one procedure, this is considered the first-line treatment modality with ESWL a second-line alternative in selected patients only (3). Either way, timeliness of access to intervention is important to prevent prolonged obstruction and potential loss of renal function (3).

As well as stone formation, cystinuria patients have the propensity to encrust indwelling stents rapidly (3, 5). If stenting an obstructing stone, swift intervention and stone clearance should be arranged, reducing the overall time a stent is left *in situ* (3). If a stent is placed at the end of the procedure, then consideration should be given to how long this is left indwelling and if the strings/tethers can be left to aid prompt removal. In our clinic, we aim to treat all patients within 2 weeks of stenting and minimize stent time to <2 weeks and use strings/ tethers where practical. Of course, leaving patients’ stent-free is preferable if safe. Patients will often know from previous experience how quickly their stents encrust, and so, this inquiry should be made.

### Renal Stones

In addition to obstructing ureteric stones, renal pelvis and calyceal stones are common in cystinuria (3). Because of the recurrent nature of these hard stones, complete clearance should be achieved when possible, but this can be challenging (3, 25, 26). The size and location of stones within the kidney will often guide the recommended treatment (5). Hounsfield units are not useful in judging the “hardness” of cystine stones, as is frequently done for calcium-containing stones, and in a large series of cystine stone formers, we found the majority of patients have Hounsfield units in the region of 400–800 (3, 27). Indeed, if Hounsfield units >1,000 are measured in a cystine stone former, then consideration of whether conversion to calcium phosphate formation has occurred as can happen in high pH ranges.

The overarching principles of endourological surgery remain the same for cystine stones as for other compositions (5, 26); however, the surgical planning and decision-making surrounding the management of them may need extra consideration, as cystine stones should not be considered as one-off events but viewed as a succession of intervention that is likely to be needed throughout the patient’s lifetime (7). In addition, the rate of stone growth may alter the surgical approach to achieve stone clearance, and certainly, the timing of the initial surgery and any follow-up procedures required should be carefully planned (28).

## Surgical Modalities

### Extracorporeal Shockwave Lithotripsy

Patients should be asked whether they have had ESWL previously and whether it had been successful. While cystine stones are often considered to be harder and resistant to ESWL, this is not always the case, and ESWL can be an effective treatment option with the right case selection (28). The literature supports that a single session of ESWL is overall less effective at achieving SFR than either URS or PCNL (29), but it is considered to be the least invasive treatment option (30). While 2 cm is usually considered the upper limit for ESWL in guidelines for general stone types (2), consensus guidelines would suggest that for lower pole cystine stones, 10 mm would be the upper limit for ESWL, and for stones 10–20 mm in other renal locations, ESWL would be a third-line option after URS and PCNL (2, 3).

Cystine stones can be further classified by the shape of their external surface into two subgroups; cystine-S, with a smooth outer layer and cystine-R, with a rough outer layer. The composition and surface shape can be detected by CT imaging. The surface type can be considered when assessing a patient for cystine stone management as those with cystine-R stones may be more amenable to successful ESWL therapy (31, 32). We previously analyzed a small cohort of our patient series and found that 47% (15/32) had stones that had responded to ESWL, and thus, for these patients, this treatment might be considered, particularly for smaller stones <1 cm (5).

### Flexible Ureteroscopy (Retrograde Intrarenal Surgery)

Globally, the rates of URS have been steadily increasing in the last 20 years, which is likely to be the result of the technological advances and improvements in scope and laser technology in this time period (33). As scopes have become smaller and more operator friendly, the ability to tackle larger, harder, and more complex renal stones using this method has increased (34). URS is often the first-line surgical approach for standard renal stones <20 mm in diameter and is an effective way of surgically managing cystine stones up to 20 mm (3, 34).

URS offers many advantages over other surgical interventions in that it is less invasive than PCNL and associated less overall complications (5). URS is associated with more favorable outcomes in terms of stone clearance and resolution than SWL. It has lower complication rates compared to PCNL for stones <2 cm (34), making it ideal for cystinuria patients who will likely require multiple lifetime procedures (3). In addition, as URS is usualy performed as a day case, this is an important consideration for patients who may require multiple procedures in their lifetime as this limits the overall impact on the disruption to their life (10, 34, 35). Recovery times are generally good, and high levels of stone clearance can be achieved in one procedure, with a relatively low retreatment rate (33). The Holmium laser fiber is effective at fragmenting all types of stones, including cystine stones (36), and leads to the characteristic sulfur smell from breakage of the disulfide bond, which is indicative that the stone contains cystine. Recently, the introduction of thulium fiber has been proposed as an effective alternative to the Holmium laser (37). This appears effective on all stone types, although larger clinical series are needed to understand the effectiveness in cystine stones.

Although considered effective, a recognized limitation of URS, and all surgical techniques to varying degrees, is the presence of retained stone fragments postsurgery and the impact this can have in cystinuria, particularly with larger stones (38). The consequence of retained stones with noncystine stones is less problematic as small fragments usually pass in the weeks or months postsurgery. In the cystinuria population, however, some literature works support that the retained stone fragment size directly correlates to further stone development and further intervention (38). Thus, whatever procedure is chosen, it should be with the aim of complete stone clearance. Thus, in larger stone burdens, it may be necessary to perform a staged “relook” procedure to completely treat large stones (3, 38).

The main disadvantage of URS can be the size of calculi urologists are able to take on, particularly those that are in difficult-to-reach locations (32). Lower pole stones, stones in angled calyces, or calyces with narrow infundibulum can sometimes be difficult to reach and fragment (32, 33). The use of ureteric access sheaths may allow larger fragments to be removed during surgery; however, they are associated with their own risk profile (39).

In the context of cystinuria, and in close discussion with the individual patient, urologists may choose to take on larger stone burdens using this approach to avoid the insult from PCNL, given the likelihood of needing multiple lifetime procedures. Consensus guidelines advocate that URS is a first-line treatment for all stones up to 10 mm in the kidney (3). For stones 10–20 mm in the lower pole, URS or PCNL may be chosen depending on lower pole anatomy and patient/surgeon preference. For stones 10–20 mm in other locations in the kidney, URS would be considered the first option with PCNL as the second line (2, 34).

### Percutaneous Nephrolithotomy

International and European guidelines recommend the use of PCNL as a first-line surgical management option for stones greater than 20 mm (2), and this is the same for cystinuria (3). PCNL traditionally requires overnight in-patient stay; however, it may also be offered as a day-case procedure for selected patients (40). In addition to large singular stones, PCNL combined with URS (ECIRS) provides excellent access to complex calculi that involve multiple areas of the collecting system and calyces (39). The improved degree of access and vision within the renal pelvis allows for larger fragments of stone to be removed and gives the greatest chance of complete stone clearance compared to other treatment modalities (40, 41). Puncturing and dilation of a tract cause trauma to the parenchyma, and complications can include bleeding, infection, and damage to nearby structures and vessels that can rarely require embolization or even nephrectomy (42).

Repeated PCNL procedures at the same location or site may compound the amount of surgical trauma to the affected kidney and result in localized scar tissue, loss of nephrons, and ultimately impact renal function over time (43). As a result, the threshold to treat stones percutaneously may be higher for cystine stone formers than it would be for other stone types, taking into consideration lifetime procedure rates and unavoidable trauma sustained to the kidney with this surgical method (3). However, due to the rate of stone formation seen in cystinuria patients, PCNL may be necessary to surgically treat large stone burdens safely and effectively. Care must be given to those with established CKD or a single kidney, both commonly seen in this cohort, to avoid further renal insult in this cohort (5).

Recently, the miniaturization of PCNL has been popularized and offers an alternative to the “standard” 24–30 Fr sheath used in conventional PCNL (40, 44). Mini-PCNL is also a good alternative to flexible ureteroscopy for larger renal stone burdens, especially when the surgeon does not feel able to clear the stone completely in one sitting. Overall, mini-PCNL is associated with less overall renal trauma than standard PCNL, with a quicker recovery time, and can result in reduced hospital stay (45). In addition, it is associated with less overall blood loss, lower transfusion rates, and fewer complications overall with the exception of infective complications (44, 45). For cystinuria patients, mini-PCNL offers a good alternative to a standard PCNL as it is associated with equivocal stone clearance results, albeit at the expense of longer operating times (45). The exact optimum or maximum stone size is unknown and will depend on surgeon preference. We have found it particularly useful in cystinuria patients, either where multiple tracts are required or with combined URS to try and ensure complete stone clearance.

Endoscopic combined intrarenal surgery (ECIRS) aims to resolve some of the limitations associated with both PCNL and URS individually by combining the two procedures (46). Both PCNL and URS are each performed simultaneously on the ipsilateral collecting system (46). ECIRS is particularly beneficial when there are stones in multiple calyces that might not be accessible through a single percutaneous tract (47). We prefer to use a single-use or “disposable” flexible ureteroscope for our ECIRS procedures due to the risk of damage to a reusable scope (48). For cystine stones, it may be advantageous to use single-use in certain cases, particularly those with high stone volume and significant stone burdens in lower poles, to avoid scope damage (49). In addition to the reduced cost of scope damage, using a single-use scope also avoids the need for a second bulky stack system to display images (48, 49).

## Conclusion

Cystinuria, and the associated formation of calculi, can be a challenging condition to manage due to recurrent pain and stone formation in a predominantly young group of patients. Compliance with both diet and fluid advice can be difficult, coupled with side effects, monitoring requirements, and availability of preventative medications to remain stone free. We have recognized these challenges and set up a dedicated cystinuria clinic that involves a multidisciplinary approach, utilizing urologists, nephrologists, radiologists, and dieticians in order to provide a one-stop clinic wherein patients are able to access these vital services, improving both compliance and the effectiveness of cystinuria management. Our cystinuria patients are routinely followed-up every 3–12 months depending on historical stone formation rates, which allows prompt detection and treatment of stone disease. The dedicated cystinuria clinic has allowed for a pragmatic and proactive approach to cystine stone management to improve patients' quality of life and overall kidney function.

 From a surgical perspective, ureteroscopy offers a first-line treatment for most patients with either renal or ureteric stones, with PCNL and mini- PCNL being reserved for particularly challenging stone burdens. ECIRS offers an opportunity to render patients with complex stone burdens stone-free in a single procedure.
